# Loss of MD1 exacerbates pressure overload-induced left ventricular structural and electrical remodelling

**DOI:** 10.1038/s41598-017-05379-w

**Published:** 2017-07-11

**Authors:** Jianye Peng, Yu Liu, Xiaoju Xiong, Congxin Huang, Yang Mei, Zhiqiang Wang, Yanhong Tang, Jing Ye, Bin Kong, Wanli Liu, Teng Wang, He Huang

**Affiliations:** 1 Department of Cardiology, Renmin Hospital of Wuhan University, Wuhan, 430060 PR China; 2 Cardiovascular Research Institute, Wuhan University, Wuhan, 430060 PR China; 3 Hubei Key Laboratory of Cardiology, Wuhan, 430060 PR China

## Abstract

Myeloid differentiation protein 1 (MD1) has been implicated in numerous pathophysiological processes, including immune regulation, obesity, insulin resistance, and inflammation. However, the role of MD1 in cardiac remodelling remains incompletely understood. We used MD1-knockout (KO) mice and their wild-type littermates to determine the functional significance of MD1 in the regulation of aortic banding (AB)-induced left ventricular (LV) structural and electrical remodelling and its underlying mechanisms. After 4 weeks of AB, MD1-KO hearts showed substantial aggravation of LV hypertrophy, fibrosis, LV dilation and dysfunction, and electrical remodelling, which resulted in overt heart failure and increased electrophysiological instability. Moreover, MD1-KO-AB cardiomyocytes showed increased diastolic sarcoplasmic reticulum (SR) Ca^2+^ leak, reduced Ca^2+^ transient amplitude and SR Ca^2+^ content, decreased SR Ca^2+^-ATPase2 expression, and increased phospholamban and Na^+^/Ca^2+^-exchanger 1 protein expression. Mechanistically, the adverse effects of MD1 deletion on LV remodelling were related to hyperactivated CaMKII signalling and increased impairment of intracellular Ca^2+^ homeostasis, whereas the increased electrophysiological instability was partly attributed to exaggerated prolongation of cardiac repolarisation, decreased action potential duration alternans threshold, and increased diastolic SR Ca^2+^ leak. Therefore, our study on MD1 could provide new therapeutic strategies for preventing/treating heart failure.

## Introduction

Hypertension is the single most critical risk factor for heart failure (HF)^1^. High blood pressure is widely recognised to induce left ventricular (LV) hypertrophy and lead initially to ventricular wall thickening and stiffening, a process which is compensatory and adaptive in nature. However, sustained pressure overload contributes to maladaptive LV remodelling, progressive LV dilatation, and cardiac dysfunction^2–4^, and this results in arrhythmias and HF^3, 5, 6^, a major underlying cause of increased cardiovascular morbidity and mortality^5, 7^. Despite being the focus of substantial research effort in recent decades, the precise pathogenesis of maladaptive LV remodelling and the mechanisms that determine how long-standing hypertrophy ultimately progresses to HF remain unclear^8^. Therefore, enhanced understanding of the factors and mechanisms that modulate pathological LV remodelling could lead to novel strategies for the treatment of HF.

Evidence gathered over the past two decades has shown that Toll-like receptor 4 (TLR4) signalling is involved in several aspects of the cardiac pathological process, such as cardiac remodelling, ischaemia/reperfusion injury, hypertension, and atherosclerosis^9–13^. Upon stimulation, TLR4 signalling ultimately activates numerous signalling pathways^14, 15^, including the MAPK pathway, NF-κB pathway, and PI3K/Akt pathway. Furthermore, a link between Ca^2+^/calmodulin-dependent kinase II (CaMKII) signalling and TLR4 signalling has been clearly demonstrated^16–18^. More importantly, therapies targeting against TLR4 have shown effectiveness in attenuating murine cardiac remodelling caused by pressure overload^19–21^. Furthermore, a recent study demonstrated that stimulation of TLR4 in rat ventricular cardiomyocytes *in vitro* promoted an electrical remodelling that led to action potential duration (APD) prolongation associated with delayed afterdepolarisation and triggered activity^22^.

The aforementioned data led us to investigate whether a naturally occurring molecule can inhibit LV remodelling and reduce the susceptibility to ventricular tachycardia (VT) during chronic pressure overload by blocking TLR4 signalling. We speculated that a favourable candidate molecule might be myeloid differentiation protein 1 (MD1), an endogenous negative modulator of TLR4 signalling^23^. MD1 is expressed predominantly in B cells, macrophages, dendritic cells, and other immune cells^23, 24^. MD1 forms a complex with radioprotective protein 105 (RP105), which is abundantly present in heart tissue. MD1-RP105 complex can directly interact with the MD2-TLR4 complex by a lateral binding, acting as physiological negative regulators of TLR4 signalling^25^. Furthermore, recent evidence suggests that MD1-RP105 complex is associated with several pathophysiological processes, including immune regulation, obesity, insulin resistance, and inflammation^26–28^. However, to the best of our knowledge, no previous study has reported whether MD1 regulates sustained pressure overload-induced LV structural and electrical remodelling.

Here, using loss-of-function approach, we discovered that pressure overload-induced LV remodelling was aggravated in MD1-knockout (KO) mice, which led to overt HF and increased electrophysiological instability. Whereas these adverse effects of MD1 deletion on LV remodelling are related to the hyperactivation of CaMKII signalling and an exaggerated impairment of intracellular Ca^2+^ homeostasis, the increased electrophysiological instability is at least partly due to an increased prolongation of cardiac repolarisation, enhanced reduction of the APD alternans threshold, and exaggerated increase of diastolic sarcoplasmic reticulum (SR) Ca^2+^ leak.

## Results

### MD1 expression is down-regulated in the left ventricles of DCM patients and in failing left ventricles in mice

**Figure 1:**
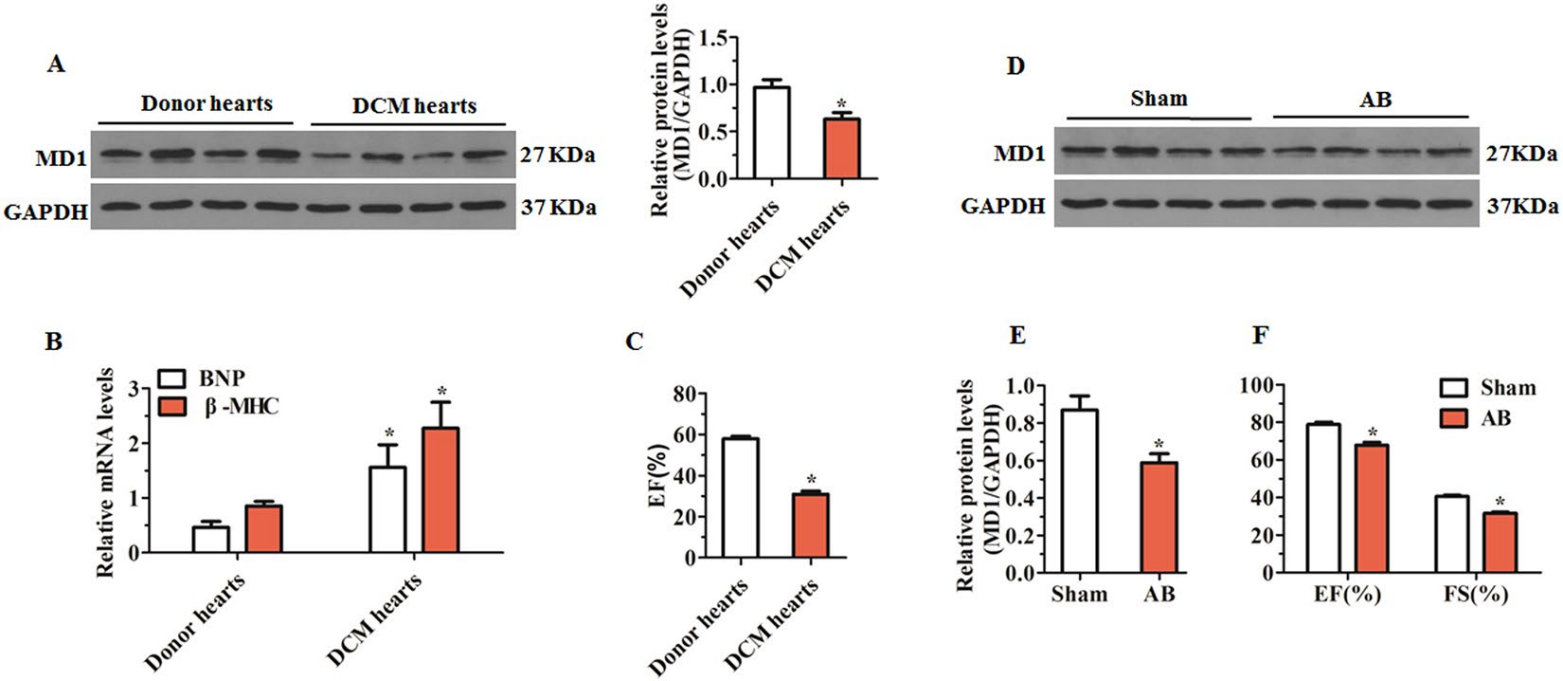
MD1 expression in left ventricles of human and mouse failing heart. (**A**) Representative western blots of MD1 in heart samples from normal donors (n = 7) and DCM patients (n = 8). (**B**) qRT-PCR analyses of BNP and β-MHC mRNA in LV tissues of normal donors (n = 7) and DCM patients (n = 8). (**C**) EF values of normal (n = 7) and DCM hearts (n = 8). (**D**,**E**) Representative western blots of MD1 and (**F**) EF/FS values in WT mouse left ventricles at 4 weeks after sham or AB operation (n = 4). *P < 0.05 vs. normal donors or shams.

To explore the correlation between MD1 and LV remodelling, we first examined MD1 expression in the failing left ventricles. Western blot analysis revealed that MD1 protein levels were significantly lower in the heart samples of DCM patients than in those of normal donors (Fig. 1A). Moreover, the diminished MD1 protein levels were accompanied with increased mRNA levels of brain natriuretic peptide (BNP) and β-myosin heavy chain (β-MHC) and decreased ejection fraction (EF) values (Fig. 1B,C). Similarly, MD1 protein levels, EF values, and fractional shortening (FS) values in wild-type (WT) mice at 4 weeks after aortic banding (AB) were significantly reduced as compared with the corresponding values in the sham-operated group (Fig. 1D–F). These results suggest that MD1 might be involved in LV remodelling.

### Deletion of MD1 exacerbates pressure overload-induced LV structural remodelling

**Figure 2:**
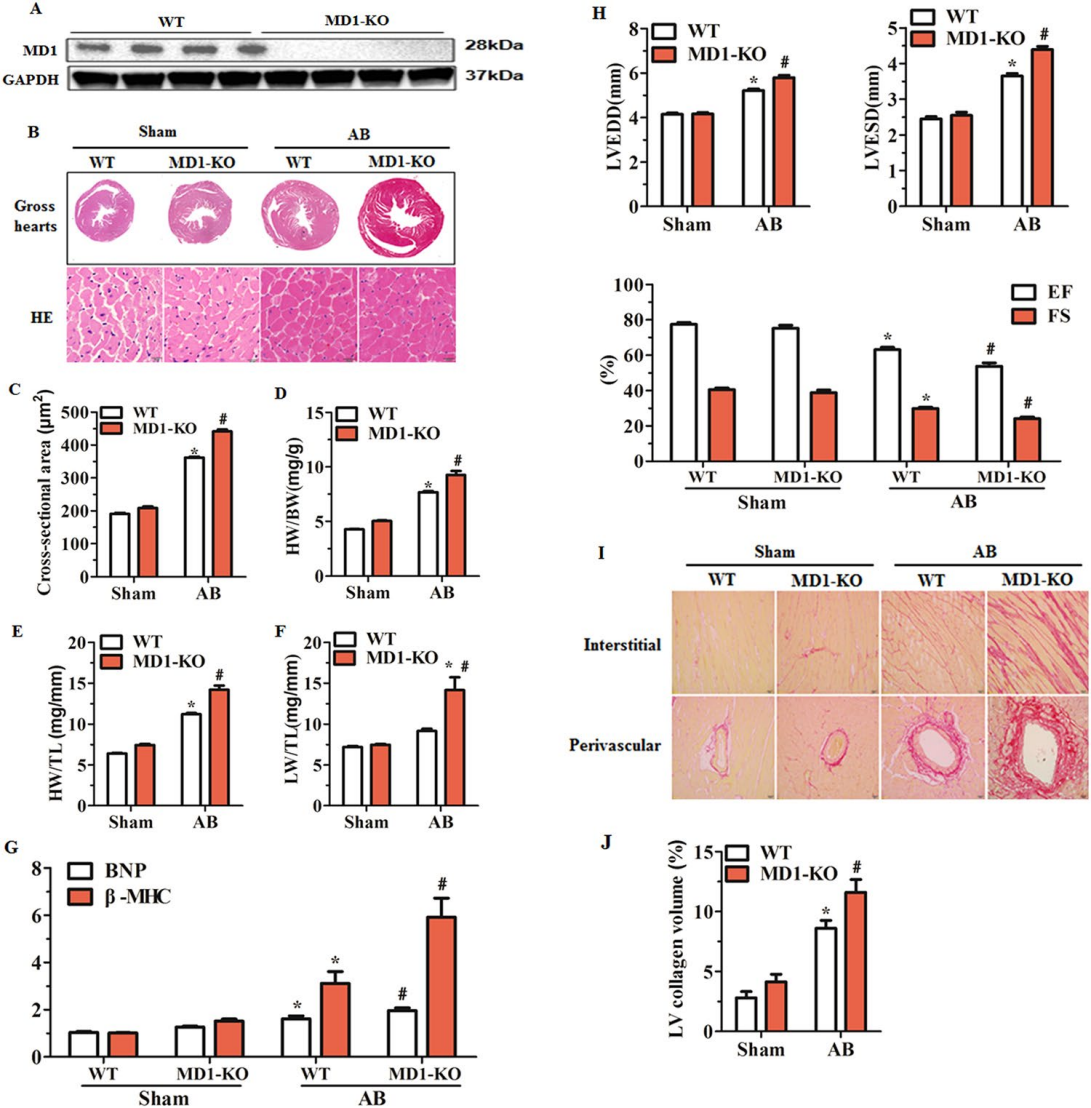
Loss of MD1 aggravates pressure overload-induced LV structural remodelling. (**A**) Representative western blots of MD1 expression in LV tissues from WT and MD1-KO mice (n = 6). (**B**) Gross hearts and H&E staining performed at 4 weeks after surgery (n = 7–8). (**C**) Statistical results of LV myocyte cross-sectional areas (n = 200+ cells). (**D**–**F**) HW/BW, HW/TL, and LW/TL values of the indicated groups (n = 13–14). (**G**) mRNA levels of the hypertrophy markers BNP and β-MHC in WT and MD1-KO left ventricles at 4 weeks after surgery, determined using qRT-PCR (n = 5). (**H**) Echocardiographic results of the indicated groups (n = 13–14). (**I**) PSR staining of histological sections prepared from LV samples of WT and MD1-KO mice at 4 weeks after surgery (n = 7–8). (**J**) Fibrotic areas from histological sections quantified using an image-analysis system (n = 26–28 fields). *P < 0.025 vs. WT-Sham, ^#^ *P* < 0.05 vs. WT-AB.

The absence of MD1 in the MD1-KO mouse heart was confirmed through Western blotting (Fig. 2A). Under basal conditions, MD1-KO mice showed no alterations in cardiac phenotype (data not shown). However, at 4 weeks after AB, MD1-KO mice exhibited a marked deterioration of LV hypertrophy as compared with their WT littermates, which was confirmed by the measurement in the KO mice of a relatively larger cardiomyocyte cross-sectional area (CSA), revealed by haematoxylin-eosin (H&E) staining (Fig. 2B,C), and higher ratios of heart weight (HW)/body weight (BW), HW/tibia length (TL), and lung weight (LW)/TL (Fig. 2D–F; Supplementary Table S1). Consistently, the mRNA levels of foetal genes (*BNP*, *β-MHC*) were markedly higher in the LV tissues of MD1-KO mice than in those of WT mice (Fig. 2G). Furthermore, MD1-KO mice exhibited exaggerated LV dilation and dysfunction, as shown by measurements of the following echocardiographic and haemodynamic parameters: LV end-diastolic diameter (LVEDD), LV end-systolic diameter (LVESD), LVEF, LVFS, LV end-systolic pressure (LVESP), and the maximum and minimum rates of LV pressure development (dp/dt max and dp/dt min, respectively) (Fig. 2H; Supplementary Table S1 and Fig. S2). Cardiac fibrosis, a major feature of maladaptive cardiac remodelling, was more prominent in MD1-KO-AB mice than in WT-AB mice (Fig. 2I,J). Collectively, these data indicate that MD1 loss exacerbates pressure overload-induced maladaptive LV structural remodelling.

### Absence of MD1 alters surface electrocardiogram (ECG) parameters during chronic pressure overload

**Figure 3:**
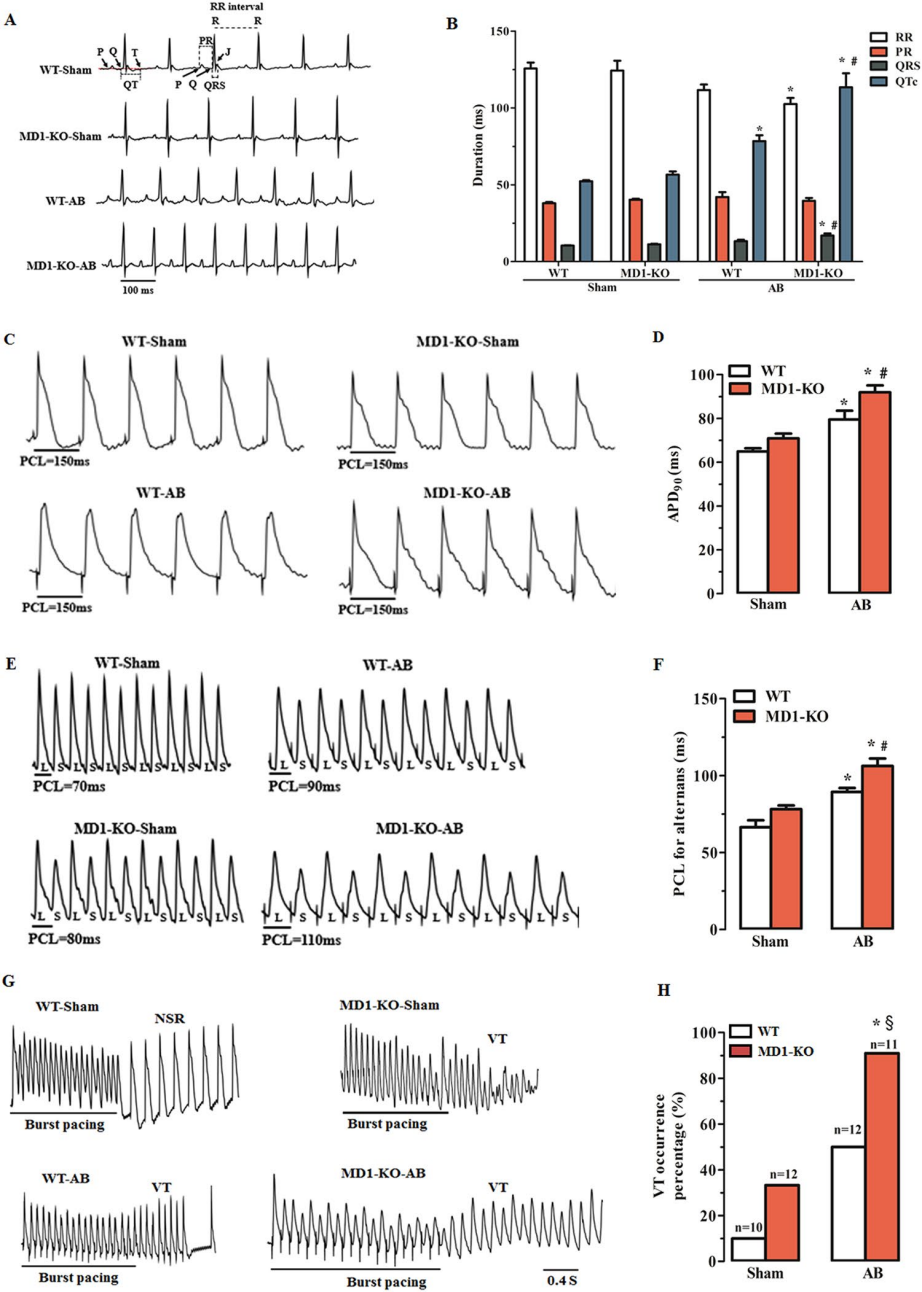
Deletion of MD1 alters surface ECG parameters and exacerbates pressure overload-induced LV electrical remodelling. (**A**) Examples of surface ECG recordings and (**B**) summary of surface ECG parameters from WT and MD1-KO mice at 4 weeks after surgery (n = 7–8). (**C**) Representative MAP recordings at a PCL of 150 ms and (**D**) statistical analysis of APD_90_ from Langendorff-perfused WT and MD1-KO hearts at 4 weeks after surgery (n = 8–9). (**E**) Representative MAP recordings of APD alternans and (**F**) statistical analysis of the threshold interval for APD alternans from the indicated groups (n = 7–8). (**G**) Examples of MAP recordings after burst pacing and (**H**) summary of VT inducibility rates in Langendorff-perfused WT and MD1-KO hearts at 4 weeks after surgery (n is indicated above the bar graphs). PCL = pacing cycle length; L = longer APD; S = shorter APD; NSR = normal sinus rhythm. *P < 0.03 vs. WT-Sham, ^§^ *P* < 0.01 vs. MD1-KO-Sham, ^#^ *P* < 0.05 vs. WT-AB.

Surface ECG (lead II) recordings were performed on mice under light anaesthesia. Mice from the groups indicated in Fig. 3 showed similar electrocardiographic PR intervals, but MD1-KO-AB mice showed shorter RR intervals and longer QRS intervals than in WT-Sham mice (Fig. 3A,B; Supplementary Table S2). The QRS intervals of MD1-KO-AB mice were significantly prolonged when compared with those of WT-AB mice (Fig. 3A,B; Supplementary Table S2). As compared with WT-Sham mice, both WT-AB and MD1-KO-AB mice exhibited prolonged QTc (corrected QT) intervals, but this prolongation was significantly higher in the KO than in the WT mice (Fig. 3A,B; Supplementary Table S2).

### Disruption of MD1 exacerbates pressure overload-induced LV electrical remodelling

Langendorff-perfused hearts and the monophasic action potential (MAP) recording technique were used to determine the changes in three electrophysiological parameters: APD_90_, threshold interval for APD alternans, and VT inducibility rate. After 4 weeks of AB, APD_90_ was markedly prolonged in both WT-AB and MD1-KO-AB mice, but the prolongation was considerably greater in the KO mice than in the WT mice (Fig. 3C,D; Supplementary Table S2). Among the four groups tested here, the changes in the threshold interval for APD alternans (Fig. 3E,F; Supplementary Table S2) were similar to the changes in APD_90_. The VT inducibility rate was potentially higher in MD1-KO-AB mice than in WT-AB mice (90.9% vs. 50%, Fig. 3G,H), although there is no significant difference (*P* *=* 0.069).

### MD1 regulates TLR4 signalling and CaMKII signalling in response to pressure overload

**Figure 4:**
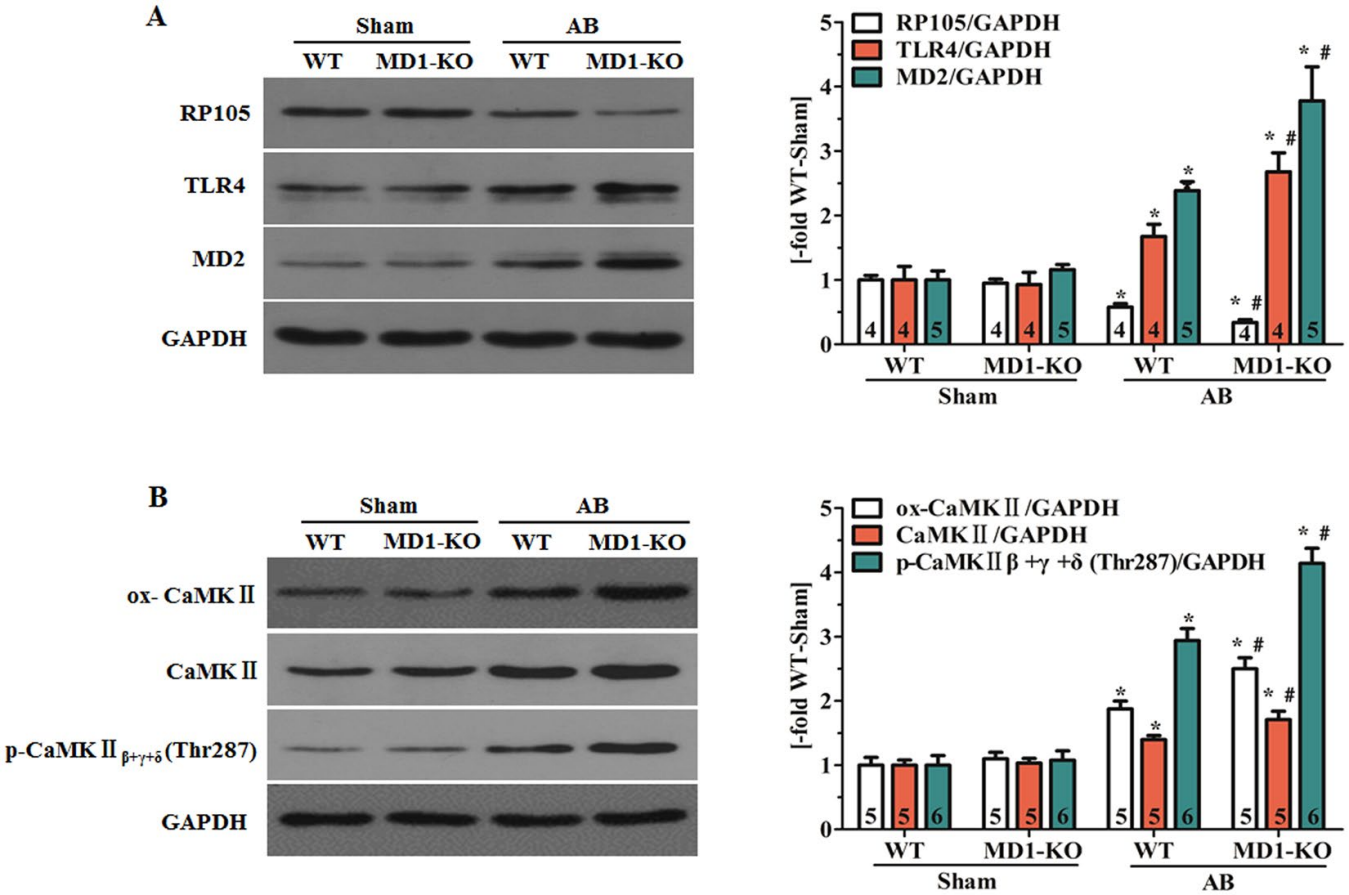
MD1 regulates TLR4 signalling and CaMKII signalling in response to pressure overload. Western blot analysis of WT and MD1-KO left-ventricle samples at 4 weeks after surgery for (**A**) RP105, MD2, and TLR4 expression; (**B**) ox-CaMKII and total CaMKII expression, and phosphorylation of CaMKIIβ + γ + δ at Thr287. Left: representative immunoblots; right: quantitative results. Data were normalised to GAPDH. Numbers of mice per group are shown inside bars. *P < 0.05 vs. WT-Sham, ^#^ *P* < 0.05 vs. WT-AB.

We applied Western blotting to investigate the effects of MD1 loss on TLR4 signalling and CaMKII signalling after 4 weeks of AB. Our results showed that a 4-week course of AB significantly decreased RP105 expression, and increased MD2 and TLR4 expression in both WT and MD1-KO LV tissues (Fig. 4A). However, the changes of RP105, MD2 and TLR4 expression were more pronounced in MD1-KO-AB mice (*P* < 0.05 vs. WT-AB; Fig. 4A), suggesting a hyperactivation of TLR4 signalling. Similarly, the CaMKII signalling was activated to a higher level in MD1-KO-AB mice than in WT-AB mice; this was revealed by the increased levels of oxidised-CaMKII (ox-CaMKII), total CaMKII expression, and phosphorylation of CaMKIIβ + γ + δ in the KO mice (Fig. 4B).

### MD1 modulates intracellular Ca^2+^ handling in response to pressure overload

**Figure 5:**
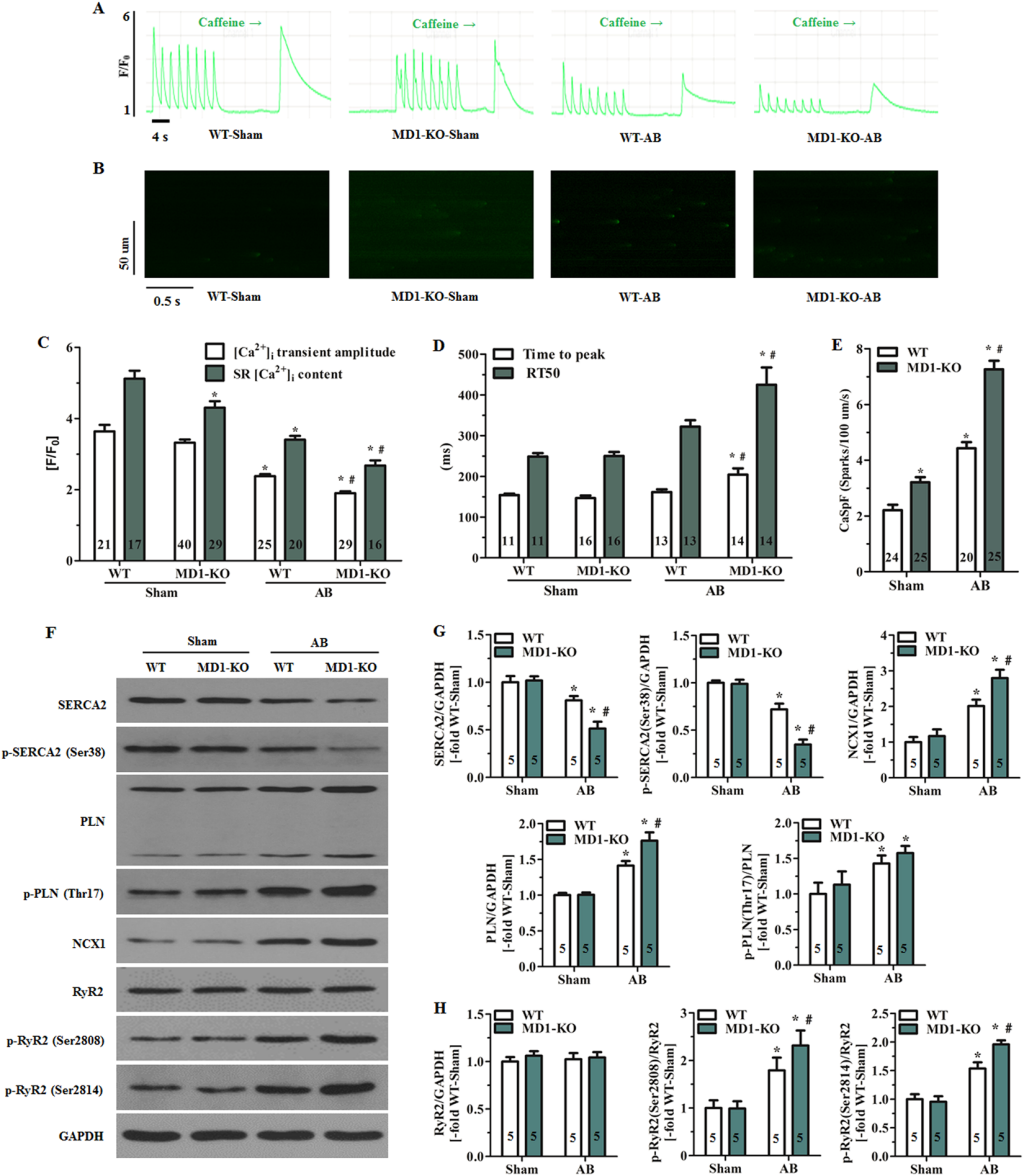
MD1 modulates intracellular Ca^2+^ handling in response to pressure overload. (**A**) Representative recordings (F/F_0_) of steady-state Ca^2+^ transients followed by rapid caffeine administration and (**B**) confocal line-scan images of mouse LV myocytes. Cells were isolated from WT and MD1-KO mice at 4 weeks after surgery. (**C**) Statistical analysis of systolic Ca^2+^ transient amplitude and SR Ca^2+^ content in the indicated groups (5–6 mice/group). (**D**) Time to peak [Ca^2+^]_i_ transient amplitude and duration of [Ca^2+^]_i_ transient decay (4–5 mice/group). RT50 = half-maximal relaxation time. (**E**) Quantification of CaSpF in the indicated groups (3–4 mice/group). (**F**) Original immunoblots of major Ca^2+^ handling proteins (including total and phosphorylated proteins). Statistical analysis of WT and MD1-KO heart samples at 4 weeks after surgery for (**G**) expression of total SERCA2, total PLN, and NCX1, phosphorylation of SERCA2 at Ser38, and PLN phosphorylation at Thr17 normalised to total PLN; (**H**) total RyR2 expression, and RyR2 phosphorylation at Ser2808 and Ser2814 normalised to total RyR2. Data were normalised to GAPDH. Numbers within columns indicate the number of LV myocytes (**C**–**E**) and heart samples (**G**,**H**). *P < 0.03 vs. WT-Sham, ^#^ *P* < 0.05 vs. WT-AB.

At baseline, the sham-operated WT and MD1-KO mice displayed similar Ca^2+^ transients in LV myocytes (Fig. 5A,C,D). However, the peak amplitude of caffeine-induced Ca^2+^ transients was lower in MD1-KO-Sham mice than WT-Sham mice (*P* < 0.05; Fig. 5A,C). After 4 weeks of AB, the LV myocytes of MD1-KO-AB mice exhibited a significantly reduced peak amplitude of Ca^2+^ transients and further reduced SR Ca^2+^ content (*P* < 0.05 vs. WT-AB; Fig. 5A,C), as well as prolonged time to peak [Ca^2+^]_i_ transient amplitude and duration of [Ca^2+^]_i_ transient decay (*P* < 0.03 vs. WT-AB; Fig. 5A,D).

We also examined whether MD1 loss increases RyR2-mediated diastolic SR Ca^2+^ leak, measured as Ca^2+^ sparks (Fig. 5B), in mouse LV myocytes. The mean Ca^2+^ spark frequency (CaSpF) values revealed that diastolic SR Ca^2+^ leak was markedly higher in MD1-KO-Sham myocytes than in WT-Sham myocytes (3.21 ± 0.18 vs. 2.21 ± 0.19 sparks/100 μm/s, *P* < 0.05; Fig. 5E). Moreover, at 4 weeks after AB, the CaSpF increase was greater in MD1-KO mice than in WT mice (7.26 ± 0.31 vs. 4.43 ± 0.22 sparks/100 μm/s, *P* < 0.001; Fig. 5E).

Under baseline conditions, the expression and phosphorylation of major Ca^2+^ handling proteins (Fig. 5F–H), and the expression of Cav1.2, calsequestrin 2 (CASQ2), and calcineurin A (Supplementary Fig. S3) were similar in WT and MD1-KO hearts. After 4 weeks of AB, SR Ca^2+^ ATPase 2 (SERCA2) expression and SERCA2 phosphorylation were drastically decreased, whereas the expression of Na^+^/Ca^2+^ exchanger 1 (NCX1) and phospholamban (PLN) were significantly increased in both MD1-KO and WT hearts, although these changes were considerably greater in MD1-KO hearts than in WT hearts (Fig. 5F,G). Moreover, PLN phosphorylation was increased, markedly and to a similar extent, in both MD1-KO-AB and WT-AB hearts (Fig. 5F,G). Phosphorylation of cardiac ryanodine receptor (RyR2) (Fig. 5F,H) and the expression of CASQ2 and calcineurin A (Supplementary Fig. S3) were markedly increased, whereas Cav1.2 expression (Supplementary Fig. S3) was significantly decreased in both WT and MD1-KO hearts by a 4-week course of AB, although these changes were significantly greater in MD1-KO-AB hearts (*P* < 0.05 vs. WT-AB).

### MD1 regulates the expression of K^+^ and Na^+^ channels in failing hearts

**Figure 6:**
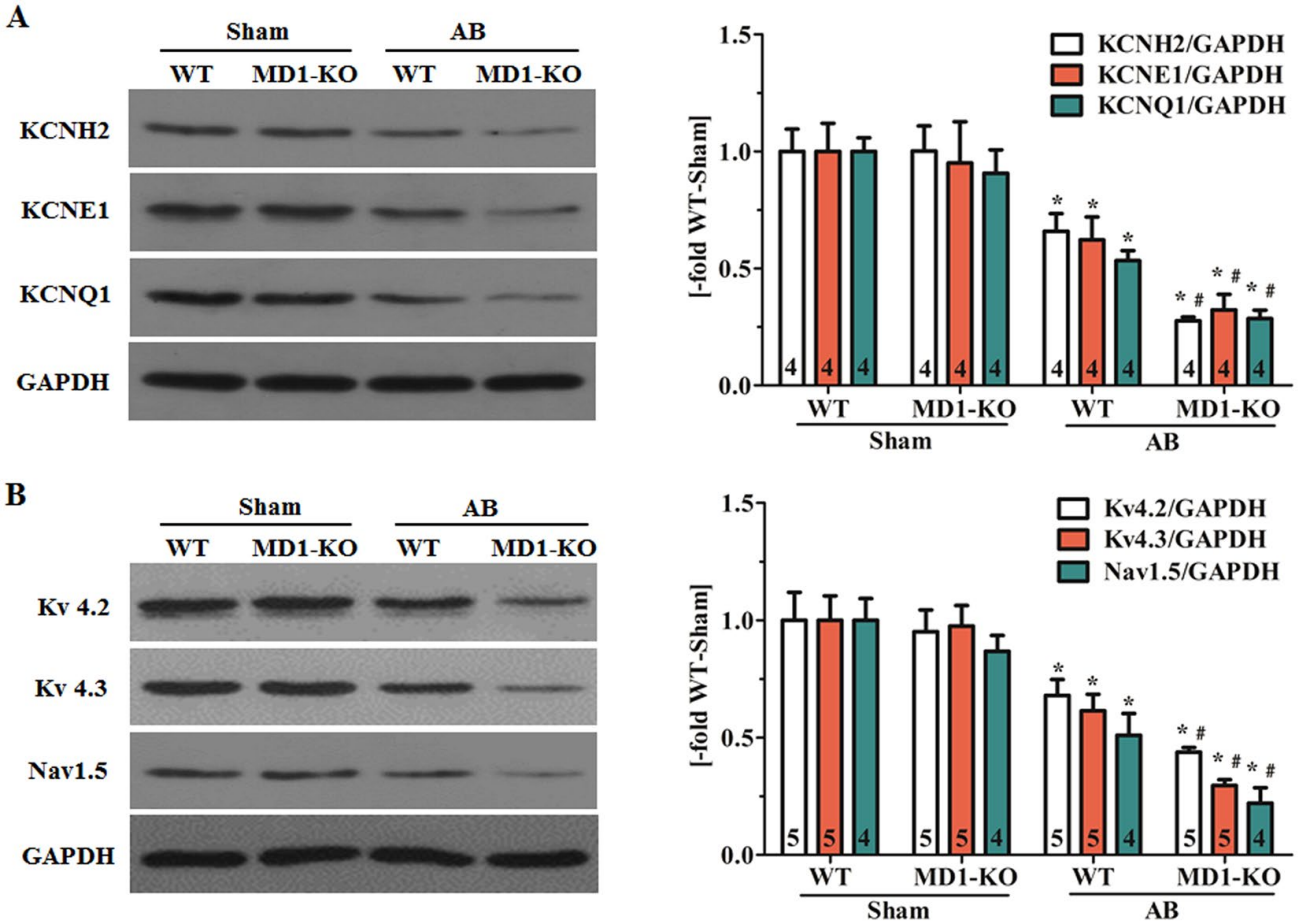
MD1 regulates the expression of K^+^ and Na^+^ channels in failing hearts. Immunoblot analysis of WT and MD1-KO left-ventricle samples at 4 weeks after surgery for (**A**) KCNH2, KCNE1 and KCNQ1 expression; (**B**) Kv4.2, Kv4.3 and Nav1.5 expression. Left: representative original immunoblots; right: quantitative results. Data were normalized to GAPDH. Numbers of mice per group are shown inside bars. *P < 0.02 vs. WT-Sham, ^#^ *P* < 0.05 vs. WT-AB.

The K^+^ and Na^+^ channels are also associated with electrical remodelling during HF, so we investigated the expression of some K^+^ and Na^+^ channels in the mouse left-ventricle samples. Our western blotting results demonstrated that after 4 weeks of AB, both WT and MD1-KO mice showed a significant reduction in protein levels of KCNH2 (Fig. 6A), KCNE1 and KCNQ1 (Fig. 6A), Kv4.2 and Kv4.3 (Fig. 6B), and Nav1.5 (Fig. 6B), which generate IKr, IKs, Ito, and INa currents, respectively. However, this reduction was more pronounced in MD1-KO-AB mice than in WT-AB mice.

## Discussion

Here, we examined the role of MD1 in pressure overload-induced LV structural and electrical remodelling by using loss-of-function strategy. Expression of MD1 is decreased in LV tissues of DCM patients and failing mouse hearts. Deletion of MD1 exaggerated LV pathological hypertrophy, fibrosis, and LV dilation and dysfunction in mice in response to persistent pressure overload, which resulted in overt HF. Moreover, in agreement with recent work^22^, we discovered that TLR4 activation resulting from a loss of MD1 expression promoted an aggravation of LV electrical remodelling, which enhanced electrophysiological instability and tended to increase the susceptibility to VT in pressure-overloaded mice. To our knowledge, this is the first report to demonstrate a critical role of MD1 in pressure overload-induced LV structural and electrical remodelling. It should be noted, however, that the real function of MD1 in the development of HF in clinical setting needs to be further determined due to the limitation of experimental conditions.

MD1 protects against pressure overload-induced LV remodelling, but the precise molecular mechanisms remain unclear. Previous studies have suggested that TLR4 activation can increase the intracellular concentration of reactive oxygen species^16–18^, which results in CaMKII oxidation and sustained activation of CaMKII signalling in stressed myocardial tissues^16, 18, 29^. Abundant evidence indicates that CaMKII is involved in cardiac remodelling and HF. Transgenic overexpression of CaMKIIδ in mouse hearts was shown to alter Ca^2+^ handling through several mechanisms and cause cardiac remodelling and diseases^30, 31^. Conversely, CaMKIIδ deletion protects against adverse ventricular remodelling after chronic pressure overload by suppressing the CaMKII/histone deacetylase signalling pathway^32, 33^, and limits the progression to HF by altering the expression of Ca^2+^-handling proteins^32^. In this study, we found that loss of MD1 led to a more pronounced activation of TLR4 signalling in mouse LVs after 4 weeks of AB. Furthermore, we found that MD1 deletion further enhanced the CaMKII signalling by CaMKII oxidation in response to chronic pressure overload. These data suggest that MD1 loss exacerbates pressure overload-triggered LV remodelling, at least partly, through hyperactivation of the CaMKII signalling.

Altered Ca^2+^ homeostasis in cardiomyocytes is a proximal trigger of stress-induced maladaptive cardiac remodelling^8^, whereas defective intracellular Ca^2+^ homeostasis is a central cause of cardiac dysfunction and arrhythmias in failing hearts^29^. RyR2 hyperphosphorylation by CaMKII causes a diastolic SR Ca^2+^ leak, which combines with reduced SERCA2-dependent Ca^2+^ uptake, contribute to a reduction in SR Ca^2+^ content and an intracellular Ca^2+^ overload and thus lead to HF^34, 35^. Our study suggests that MD1 loss increases SR Ca^2+^ leak, and decreases SR Ca^2+^ content and Ca^2+^ transient amplitudes in failing LV myocytes, but this wasn’t the case with the reduction of peak L-type Ca^2+^ channel current (I_Ca,L_) density (Supplementary Fig. S4A–C). The reduced SR Ca^2+^ content, a major cause for the severe cardiac dysfunction in MD1-KO-AB mice, was associated with increased SR Ca^2+^ leak and diminished SERCA2-dependent Ca^2+^ uptake, and was likely associated with increased expression of NCX1. Unexpectedly, the expression of CASQ2 was also increased. We speculate that this might represent a compensatory response to the increased SR Ca^2+^ leak: it might contribute to RyR2 deactivation and refractoriness early after Ca^2+^ release and to the reduced SR Ca^2+^ buffering^36^. In agreement with recent studies^37–39^, our study also suggested that exaggerated RyR2 dysfunction led to a hyperactivation of calcineurin/NFAT signalling pathway, which has been implicated in stress-induced pathological remodelling of the heart^6, 8^.

Prolongation of cardiac repolarisation is widely recognised to increase the risk of malignant ventricular arrhythmias (VAs) and sudden cardiac death^40^. QTc interval is a cardiac repolarisation marker and it corresponds to APD at the cellular scale; when QTc interval and APD are prolonged, they become pro-arrhythmic factors^40^. Consistent with these data, MD1-KO-AB hearts demonstrated prolonged QTc interval and APD, and a tendency of increasing the susceptibility to VT when compared with WT-AB hearts in this study. Moreover, our result agrees with the findings of a recent study suggesting that activation of cardiac TLR4 by lipopolysaccharide markedly increases APD^22^. Cardiac repolarization is mainly controlled by K^+^ currents, and CaMKII overactivity can impair K^+^ channel function and thereby prolong APD^29, 41^. Therefore, we examined the effect of MD1 deletion on protein expression of KCNH2, KCNE1 and KCNQ1, Kv4.2 and Kv4.3, which generate Ikr, Iks and Ito currents, respectively. Our results suggest that the APD prolongation observed in isolated MD1-KO-AB hearts might partly be through regulating cardiac K^+^ channels. However, the direct effect of MD1 deletion on Ikr, Iks and Ito currents in failing hearts needs to be further determined.

T-wave alternans (TWA) is a risk marker of cardiac repolarisation that has been associated with VAs^40, 42^, and interventions that eliminate TWA might prevent VT and sudden death^42^. TWA corresponds to APD alternans at the cellular level^40, 43^. Therefore, any factor that decreases the APD alternans threshold might induce VT. Our results showed that MD1 loss decreased the APD alternans threshold in isolated pressure-overload hearts, which suggests that reduction of the APD alternans threshold is one of the mechanisms underlying the MD1 deletion-elicited arrhythmogenic potential in response to pressure overload. A recent literature review revealed that increased SR Ca^2+^ leak through RyR2 channels and/or decreased SERCA pump activity contributes to alternans^43^. Consistent with this review’s finding, our study has shown that SERCA2 expression and phosphorylation levels were lower but RyR2 phosphorylation was higher in MD1-KO-AB mice than in WT-AB mice. In addition, alternans can be caused by instabilities originating from voltage and is influenced by I_Na_ in electrically remodelled diseased hearts^43^. I_Na_ is affected by CaMKII activity^29, 41^. Although I_Na_ might be reduced as Nav1.5 expression was demonstrated to be markedly decreased in MD1-KO-AB mice in this study, but the activation and kinetic properties of I_Na_ remain to be explored in future work.

RyR2 hyperphosphorylation due to CaMKII overactivity is widely accepted to lead to increases in diastolic SR Ca^2+^ leak, which can cause Ca^2+^ waves and activate an arrhythmogenic inward Na^+^/Ca^2+^ exchange current, causing delayed afterdepolarisations (DADs) and triggering arrhythmias^29, 44–46^. Here, MD1 loss enhanced the increase in CaMKII activity and CaMKII-mediated RyR2 phosphorylation in response to chronic pressure overload. Moreover, the LV myocytes isolated from MD1-KO-AB mice exhibited an increased arrhythmogenic potential *in vitro* as compared with WT-AB mice, including enhanced diastolic SR Ca^2+^ leak, reduced SERCA2 pump activity, and increased NCX1 expression. Growing evidence indicates that increased SR Ca^2+^ loading is necessary for enhanced RyR2 Ca^2+^ leak to produce arrhythmia^47^, but no direct evidence is available to demonstrate that spontaneous Ca^2+^ sparks arise only when a specific SR cistern becomes ‘overloaded’ and that a lower-than-physiological concentration of Ca^2+^ in the SR does not affect Ca^2+^ spark production^48^. Collectively, these data indicate that the enhanced diastolic SR Ca^2+^ leak is likely sufficient to contribute to the electrophysiological instability, although the occurrence of spontaneous Ca^2+^ waves, and DADs needs to be further investigated.

This work has limitations in the study of cardiomyocyte Ca^2+^ handling. (1) The analytical results of I_Ca,L_ inactivation (Supplementary Fig. S4D,E) was not consistent with previous reports that I_Ca,L_ decay is slowed in HF^49–51^. Fitting I_Ca,L_ decay using a double exponential equation to calculate the fast and slow decay of I_Ca,L_ would be better to analyse I_Ca,L_ decay^52^, and to know whether at the end of the stimulation pulse there is some residual current. (2) The increased NCX1 expression as a factor for the decreased SR Ca^2+^ content in MD1-KO-AB mice was not confirmed. Although enhanced NCX activity could decrease SR Ca^2+^ content in HF^29, 35, 53^, and increased NCX expression was proved to be associated with the decreased SR Ca^2+^ content in right atrial myocytes isolated from the patients with chronic atrial fibrillation^54^, additional analysis are required to calculate the contribution of NCX to Ca^2+^ removal. The way to do that is to fit exponential decay both on the systolic and caffeine transients and calculate rate constants of decay, as previously described^54, 55^. Due to technical problems and insufficient experience, we couldn’t completely solve these in a short time.

In summary, the present study indicates that MD1 loss exacerbates LV structural and electrical remodelling in response to chronic pressure overload, resulting in overt HF and increased electrophysiological instability. Mechanistically, the adverse effects of MD1 deletion on LV remodelling are associated with hyperactivated CaMKII signalling and increased impairment of intracellular Ca^2+^ homeostasis. The increased electrophysiological instability is partly due to aggravated electrical remodelling, including prolonged cardiac repolarisation, decreased APD alternans threshold, and increased diastolic SR Ca^2+^ leak. These findings suggest that endogenous MD1 protects against LV structural and electrical remodelling during long-standing pressure overload. Therefore, MD1 upregulation in the heart might serve as a new therapeutic strategy for the treatment of HF.

## Methods

### Human heart samples

Samples of human failing hearts were collected from the left ventricles of DCM patients undergoing heart transplants. Control samples were obtained from the left ventricles of normal heart donors who had died as a result of accidents, and whose hearts were not used for transplantation due to non-cardiac reasons. After excision, all specimens were immediately flash-frozen in liquid nitrogen and stored at −80 °C for biochemical analysis. Written informed consent was obtained from the family of prospective heart donors. The study was approved by Renmin Hospital of Wuhan University Review Board and was conducted in accordance with the Declaration of Helsinki.

### Mice

Male MD1-KO mice (C57BL/6 background, purchased from RIKEN, RBRC02386) and their WT littermates aged 8–10 weeks were used for experiments. All animals were housed in a 12/12-h light/dark cycle and provided food and water ad libitum. Mice were genotyped by means of PCR performed using the protocol provided by RIKEN (Supplementary Methods and Fig. S1). All animal experiments were performed according to the Guide for the Care and Use of Laboratory Animals published by the US National Institutes of Health (Publication No. 85-23, revised 1996) and approved by the Animal Care and Use Committee of Renmin Hospital of Wuhan University.

### Animal model

All surgeries and subsequent analyses were performed in a blinded fashion for all groups. AB surgery and sham operation were performed as described previously^56, 57^. Doppler analysis was performed to ensure the induction of adequate constriction of the aorta. LV internal diameter and wall thickness were evaluated through echocardiography at various times after surgery. At the end of these procedures, some of the mice of the tested groups were sacrificed using an overdose of pentobarbital sodium (150 mg/kg, intraperitoneal injection), and ratios of HW/BW (mg/g), HW/TL (mg/mm), and LW/TL (mg/mm) of the sacrificed mice from the different groups were assessed. The LV tissues from each heart were dissected, snap-frozen in liquid nitrogen, and stored at −80 °C for biochemical studies.

### Pressure-volume relationship measurements and echocardiography

We performed pressure-volume relationship measurements and echocardiography as described previously^56, 57^ to obtain these values: LVEF, LVFS, LVEDD, LVESD, LVESP, interventricular septum diameter in diastole and systole (IVSd and IVSs, respectively), LV posterior wall diameter in diastole and systole (LVPWd and LVPWs, respectively), and dp/dt max and dp/dt min.

### Histological analysis

Hearts were excised and washed with saline solution, arrested in diastole with 10% KCl, fixed in 4% paraformaldehyde solution, and embedded in paraffin. The paraffin-embedded hearts were sectioned transversely at the level of the LV papillary muscles. Several slices (4–5 mm thick) of heart were prepared and stained with H&E for morphometric analysis or picrosirius red (PSR) for evaluation of myocardial fibrosis. All micrographs were acquired using a high-resolution optical microscope. Image-Pro Plus 6.0 software was used to determine the CSA of cardiomyocytes and the volume of LV collagen deposition.

### Quantitative real-time PCR (qRT-PCR)

Total RNA from human or mouse LV tissues was extracted and reverse transcribed to generate cDNAs; qRT-PCR was performed by using an ABI-PRISM 7900 Sequence Detection System with the SYBR Color qPCR Master Mix (Vazyme Biotech Co, Nanjing, China). Gene expression was normalised relative to the housekeeping gene *GAPDH*, and data were analysed according to the 2^−∆∆Ct^ method^58^. The method details are described in Supplementary Methods.

### Western blotting

Total proteins were extracted from frozen LV samples, and a BCA Protein Assay Kit (Beyotime Biotechnology, P0010) was used to determine protein concentrations. Proteins (40 μg) were separated using SDS-PAGE and transferred onto polyvinylidene difluoride membranes, which were then incubated with primary antibodies overnight at 4 °C and subsequently with secondary antibodies for 2 h at room temperature. Chemiluminescent detection was performed using the SuperSignal West Pico Chemiluminescent Substrate (Thermo Scientific, NCI5079). Additional details are presented in Supplementary Methods.

### Surface ECG recording and analysis

Mice were lightly anaesthetised using inhaled isoflurane (1.5% isoflurane in 98% O_2_). Surface electrodes were placed subcutaneously and the surface-lead ECG (lead II) was recorded. Data were analysed off-line using LabChart 7 Pro (AD Instruments). To correct for heart rate, the QTc interval was calculated using Bazett’s formula^59^: QTc = QT/(RR/100)^1/2^. Supplementary Methods contains additional details.

### Preparation of Langendorff-perfused hearts and MAP recording

Langendorff-perfused hearts were prepared according to published methods^60^. Epicardial MAP was recorded from the LV anterior free wall by using a custom-made MAP electrode, constructed from two 0.25-mm Teflon-coated silver wires (99.99% purity). The paired platinum stimulating electrode was positioned on the basal surface of the right ventricle, and it delivered regular pacing. Method details are described in Supplementary Methods.

### Protocols used for electrical stimulation of isolated hearts

S1-S1 pacing was used for determining APD_90_ and APD alternans threshold; pacing was performed with a series of pulse trains at a regular pacing cycle length (PCL) for 30 s, and then interrupted for at least 30 s to minimise pacing memory effects. The PCL was shortened from 150 to 100 ms in 10-ms steps and then to 50 ms in 5-ms steps. The APD_90_ at the PCL of 150 ms was defied as the average 90% repolarisation time for 6–8 successive MAPs. Alternans was judged to have occurred when alternate APDs differed by 5% over at least 10 beats^61^. The APDs were measured at 90% repolarisation and the longest PCL-induced APD_90_ alternans was defined as the APD alternans threshold interval. To test for VT inducibility, burst pacing at cycle lengths of 100–50 ms or 2:1 capture occurrence was used^62, 63^. Unlike in previous studies, we applied a pacing train of 20 stimuli. VT was defined as the occurrence of at least 4 consecutive ventricular waveforms after the last paced beat.

### Preparation of mouse LV myocytes

Single LV myocytes were isolated from the mice by using collagenase type II digestion. Freshly isolated LV myocytes were stored at room temperature until use. Only rod-shaped myocytes showing clear striations were studied, and experiments were performed at room temperature (20 °C–25 °C) within 6 h after cell isolation. Experimental details are presented in Supplementary Methods.

### Patch-clamp recording

Whole-cell membrane currents were obtained and assessed by using an EPC-9 patch-clamp amplifier (HEKA Electronik, Lambrecht, Germany) in the whole-cell mode and Pulse/Pulsefit software (HEKA Electronik). Supplementary Methods contains the experimental details.

### Measurements of Ca^2+^ transients and SR Ca^2+^ content

Isolated cardiomyocytes were loaded with the Ca^2+^ indicator Fluo-4 AM (10 μmol/L; Molecular Probes, Invitrogen, F14217) in Tyrode’s solution (mmol/L: NaCl 135; KCl 5.4; CaCl_2_ 1.8; MgCl_2_ 1; NaH_2_PO_4_ 0.33; HEPES 10; glucose 10; pH adjusted to 7.35 with NaOH) at 37 °C for 30 min; cells were suspended every 5 min during incubation. The loaded cells were washed for 15 min for de-esterification, superfused for 10 min with dye-free Tyrode’s solution, and then transferred to a chamber equipped with a pair of parallel platinum electrodes. The chamber was placed on a Leica AF6000 fluorescence microscope (Leica Microsystems Inc, Germany). Systolic Ca^2+^ transients were recorded in steady-state conditions under constant field stimulation (8 pulses at 0.5 Hz, 10 V). We evaluated the average time to peak Ca^2+^ transient amplitude and duration of Ca^2+^ transient decay of a tested cardiomyocyte by measuring 4 consecutive and well performed Ca^2+^ transients during field stimulation. To assess SR Ca^2+^ content, the amplitude of caffeine-induced Ca^2+^ transients was measured; at 4 s after the stimulation was stopped, 10 mmol/L caffeine was applied directly onto the cells, which led to an immediate and complete SR Ca^2+^ release. The fluorescence intensity of Fluo-4 (measured at 526 nm upon excitation at 488 nm) was recorded and the Ca^2+^ transient amplitude (F/F_0_) and duration were assessed, both by using the Leica AF6000 Modular system.

### Ca^2+^ spark measurements

Loaded cardiomyocytes were prepared as described above and transferred to the recording chamber, which was placed on a Leica TCS SP8 STED 3X laser-scanning confocal microscope (Leica Microsystems Inc.). Fluo-4 was excited using a 488-nm argon-ion laser and the emitted fluorescence was measured at 526 nm. Diastolic Ca^2+^ sparks of the loaded cardiomyocytes were recorded at resting conditions after repetitive field stimulation (8 pulses at 0.5 Hz, 10 V). Ca^2+^ sparks were analysed using SparkMaster and Image J^64^. The mean CaSpF of each recorded cell was expressed as sparks/100 μm/s.

### Statistical analysis

Statistical analysis was performed using SPSS or GraphPad Prism software. Continuous variables are shown as means ± SEM and were evaluated with Student’s 2-tailed unpaired *t* test or two-way ANOVA followed by Bonferroni post hoc test. Categorical data are expressed as percentages and were analysed using Fisher exact test. *P* < 0.05 was considered statistically significant.

## Electronic supplementary material

Supplementary Materials
