# Supplementary material for: Loss of MD1 exacerbates pressure overload-induced left ventricular structural and electrical remodelling
Source: Sci Rep. 2017 Jul 11;7:5116. doi: 10.1038/s41598-017-05379-w (PMC5505950; doi:10.1038/s41598-017-05379-w)
Supplement: Supplementary file 1 — Supplementary Materials [file 41598_2017_5379_MOESM1_ESM.pdf]

Supplementary Materials

Loss of MD1 exacerbates pressure overload-induced left ventricular structural and electrical remodelling

Jianye Peng[1,2,3], Yu Liu[1,2,3], Xiaoju Xiong[1,2,3], Congxin Huang[1,2,3], Yang Mei[1,2,3], Zhiqiang Wang[1,2,3], Yanhong Tang[1,2,3], Jing Ye[1,2,3], Bin Kong[1,2,3],Wanli Liu[1,2,3], Teng Wang[1,2,3], He Huang[1,2,3]*

[1]Department of Cardiology, Renmin Hospital of Wuhan University, Wuhan 430060, PR China.

[2]Cardiovascular Research Institute, Wuhan University, Wuhan 430060, PR China.

[3]Hubei Key Laboratory of Cardiology, Wuhan 430060, PR China.

Jianye Peng and Yu Liu are co-first authors. *Corresponding author: He Huang, MD, PhD, FACC, FESC, FEHRA, Department of Cardiology, Renmin Hospital of Wuhan University; Cardiovascular Research Institute, Wuhan University; Hubei Key Laboratory of Cardiology, Jiefang Road 238, Wuhan 430060, PR China. Tel/Fax: 86-27-88041911. E-mail: huanghe1977@whu.edu.cn

Supplementary methods

Mouse genotyping protocol

Mice were genotyped by PCR using a mixture of three primers (forward, 5′-GCCTGGCAGAATATTCA-GCTAC-3′; reverse, 5′-CACAGCTCTGGTAGACTACTTC-3′; neo, 5′-CGGCCACAGTCGATGAATCC-3′). The PCR conditions consisted of 94°C for 3 min, 30 cycles of 94°C for 30 s each, 58°C for 30 s, and 72°C for 3 min, followed by 72°C for 5 min.

Quantitative Real-time PCR (qRT-PCR)

Total RNA was extracted from frozen, pulverized human or mouse left ventricular (LV) tissues using Trizol Reagent (Aidlab, Beijing, China) according to the manufacturer's protocol. RNA concentration and quality were determined by spectrophotometry. cDNA fragments were synthesized by reverse transcription using the cDNA Synthesis Kit (GeneCopoeia). The qRT-PCR was carried out by using ABI-PRISM 7900 Sequence Detection system with SYBR Color qPCR Master Mix (Vazyme Biotech Co, Nanjing, China). Primers used for the detection of human brain natriuretic peptide (BNP) and β-myosin heavy chain (β-MHC) were as follows: BNP: forward, 5′-TCAGCCTCGGACTTGGAAACG-3′; BNP: reverse, 5′-CGCAGGGTGTAGAGGACCATTTT-3′; β-MHC: forward, 5′-CGTTCTGTCAACGACCTCAC-3′; β-MHC: reverse, 5′-TTCTTCGCCTTAACCTCCTC-3′. Primers used for the detection of mouse BNP and β-MHC were as follows: BNP: forward, 5′-GAGGTCACTCCTATCCTCTGG-3′; BNP: reverse, 5′-GCCATTTCCTCCGA-CTTTTCTC-3′; β-MHC: forward, 5′-CCGAGTCCCAGGTCAACAA-3′; β-MHC: reverse, 5′-CTTCACGGGCAC-CCTTGGA-3′. Gene expression was normalized to the housekeeping gene *GAPDH*. Data were analyzed according to the $2^{-\Delta\Delta Ct}$ method[1].

Western Blotting

Western blot analyses were performed on total protein extracts prepared from human or mouse LV tissues. Tissues were pulverized, and resuspended in ice-cold lysis buffer (Beyotime Biotechnology, P0013B) containing PMSF (Beyotime Biotechnology, ST506) and Phosphatase Inhibitor Cocktail Tablets (Roche, 04906845001), all of these reagents were used according to manufacturers' protocols. After 30 min incubation with slow rotation at 4°C, the insoluble fraction was removed by centrifugation at 12,000 rpm for 5 min at 4°C, the supernatant was transferred into tubes for western analysis. Quantification of total proteins in each sample was obtained by using the BCA Protein Assay Kit (Beyotime Biotechnology, P0010).

Forty micrograms of lysate aliquots were size-fractionated on 8% to 15% SDS-polyacrylamide gels, then the resolved gels were electro-transferred on PVDF membranes (Millipore). Antibodies against MD1 (1:200; Santa Cruz, sc-390613), MD1 (1:800; Abcam, ab45424), RP105 (1:1,000; Abcam, ab184956), TLR4 (1:1,000; ImmunoWay, YT0744), MD2 (1:1,000; Bioworld, BS7308), RyR2 (1:1,000; Merck Chemicon, AB9080), phospho-Ser2814-RyR2 (1:5,000; Badrilla, A010-31), phospho-Ser2808-RyR2 (1:5,000; Abcam, ab59225), PLN (1:1,000; Cell Signaling, 8495), phospho-Thr17-PLN (1:5,000; Badrilla,A010-13), Cav1.2 (1:800; Thermo Scientific, PA5-23013), NCX1 (1:4,000; Abcam, ab177952), SERCA2 (1:50,000; Abcam, ab150435), phospho-Ser38-SERCA2 (1:1,000; Badrilla, A010-25AP), CASQ2 (1:3,000; Abcam, ab108289), CaMK II (1:400; Santa Cruz, sc-9035), oxidized-CaMKII (1:1,000; GeneTex, GTX36254), phospho-Thr287-CaMKIIβ+γ+δ (1:800; Thermo Scientific, PA5-37833), Calcineurin A (1:20,000; Abcam, ab109412), Kv 4.2 (1:1000; Sigma Aldrich, SAB5200070), Kv 4.3 (1:1000; Sigma Aldrich, SAB5200076), KCNH2 (1:1,000; Bioworld, BS7461), KCNE1 (1:1,000; Bioworld, BS6212), KCNQ1

(1:1,000; Bioworld, BS6923), Nav1.5 (1:2,000; Abcam, ab56240), GAPDH (1:5,000; Cell Signaling, 5174S) were used to probed with the membranes at 4°C overnight or at room temperature for 4 h.
After washing, the membranes were incubated with a goat anti-rabbit or anti-mouse horseradish peroxidase-conjugated secondary antibody (1:5,000–1:10,000, Millipore). Membranes were incubated with the SuperSignal West Pico Chemiluminescent Substrate (Thermo Scientific, NCI5079), and signal was exposed to x-ray film (Kodak). Films were scanned and the densities of specific bands were determined using Image J software. Protein-signal densities were normalized to the corresponding GAPDH-signal densities, the phosphorylation-signal densities were normalized to the corresponding total protein-signal densities when necessary.

Surface Electrocardiogram (ECG) recording and analysis

Surface-lead ECG (lead II) recording was performed on mice under light anesthesia. Mice were lightly anesthetized by inhaled isoflurane (1.5% isoflurane in 98% O_2) and were positioned on a custom-made ECG recording platform, body temperature was maintained at 37°C by use of a heating pad controlled by a temperature controller (World Precision Instruments). The Ag/AgCl gel-coated ECG electrodes were placed subcutaneous and connected to a standard 6-lead ECG amplifier module (AD Instruments, Australia), which included high and low pass filters (set to 0.05 Hz and 1kHz, respectively) and a gain selection device (set to 1000-fold). ECG Signals were continuously recorded at 1 kHz sampling rate using the data acquisition system (AD Instruments) for 30 min.
Data were analyzed off-line using LabChart 7 Pro (AD Instruments). After scanning the ECG signals for rhythm disorders and noise, a stable periods of 5 min were analyzed to determine RR, PR, QRS and corrected QT (QTc) intervals. The RR interval was determined automatically by averaging the time between two consecutive RR waves. The PR interval was measured from the beginning of the P wave to the beginning of the QRS complex. QRS duration was measured from the first deflection of the Q wave (or the R wave when the Q wave was absent) to the point where the negative part of the S wave returned to the isoelectric line. The QT interval was measured from the beginning of the QRS complex to the end of the T wave. To correct for heart rate, QTc interval was calculated with Bazett's formula[2]: $QTc = QT/(RR/100)^{1/2}$.

Preparation of Langendorff-perfused hearts

The isolated Langendorff-perfused hearts were prepared according to published methods[3]. Mice were heparinized by heparin sodium (100U, intraperitoneal injection) for 10 min, then anesthetized with sodium pentobarbital (50 mg/kg, intraperitoneal injection) and adequacy of anesthesia was monitored by testing the pedal reflex. Hearts were quickly excised and transferred to oxygenated and ice-cold Tyrode's solution (mmol/L: NaCl 135; KCl 5.4; $CaCl_2$ 1.8; $MgCl_2$ 1; NaH_2PO_4 0.33; HEPES 10; glucose 10; pH adjusted to 7.35 with NaOH). The ascending aorta was identified and cannulated with a tailor-made 21-gauge cannula that had been prefilled with ice-cold buffer, make sure the terminal of the gauge cannula was located above of the aortic root. The aorta was secured onto the cannula with a micro-aneurysm clip. The heart was then rapidly transferred and fixed to the langendorff-perfusion system (AD Instruments). The oxygenated perfusate was passed through the pipeline and warmed to 37°C by a water jacket and circulator, then through the aorta at 2–3 ml/min by a peristaltic pump (AD Instruments, Australia).The perfusion pressure was maintained at 80-100mmHg. By this way, the coronary arteries were perfused with oxygenated Tyrode's solution passing through the aorta. After the initiation of perfusion, hearts regained a pink color and spontaneous rhythmic contractions. All the

isolated hearts were perfused for more than 10 min before further experiments. The hearts that did not recover to regular spontaneous rhythm or had irreversible myocardial ischemia were discarded.

Monophasic action potential (MAP) recording

MAP was recorded from the epicardium of the LV anterior free wall using a custom-made MAP electrode, constructed from two 0.25 mm Teflon-coated silver wire (99.99% purity) which were twist together and galvanically chlorided to eliminate DC offset. The paired platinum stimulating electrode was positioned on the basal surface of right ventricle and delivered regular pacing. MAPs were amplified with an amplifier and band pass filtered between 0.3 Hz and 1 kHz. MAP waveforms were analyzed using LabChart 7 Pro software.

Preparation of mouse LV myocytes

Mice were heparinized and anesthetized as described above, the hearts were quickly removed and placed into a cold and oxygenated Ca^{2+}-free Tyrode's solution containing (in mmol/L) NaCl 135, KCl 5.4, $MgCl_2$ 1, NaH_2PO_4 0.33, HEPES 10, glucose 10, adjusted to pH 7.35. The ascending aorta was cannulated onto the langendorff-perfusion system at a 2–3 ml/min flow rate of perfusion. The heart was perfused with oxygenated Ca^{2+}-free Tyrode's solution at 37°C for 5–10 min to flush away any blood inside the heart. For enzymatic dissociation, the heart was perfused with oxygenated Ca^{2+}-free Tyrode's solution containing collagenase type II (0.3–0.5 mg/ml; Sigma Aldrich, C6885) and BSA (2 mg/ml; Roche Diagnostics, 10735086001) for 10–15 min at 37°C. Then the heart was removed and placed into a dish containing Tyrode's solution supplemented with 0.1 mmol/L $CaCl_2$ and 2 mg/ml BSA. The left ventricle was separated from the heart, cut into small pieces, and triturated with a pipette to disperse the myocytes. Ventricular myocytes were filtered on gauze and sedimented by gravity for 10 min. The supernatant was removed, and cells were suspended in Tyrode's solution containing 0.3 mmol/L $CaCl_2$ and 2 mg/ml BSA. The procedure was repeated once, and cells were suspended in Tyrode's solution containing 0.5 mmol/L $CaCl_2$ and 2 mg/ml BSA. Freshly isolated LV myocytes were stored at room temperature until use. Only rod-shaped myocytes showing clear striations were studied, and experiments were performed at room temperature (20–25°C) within 6 h after cell isolation.

Patch-clamp recording

The whole-cell patch-clamp technique was used to record L-type Ca^{2+} channel current ($I_{Ca,\,L}$). Patch electrodes were pulled from borosilicate glass (Sutter Instrument, BF150-86-10) using six-stage pulling on a Flaming/Brown Micropipette puller (Sutter Instrument, Model P-97). The fire-polished electrodes had a resistance of 4 to 8 MΩ when filled with internal solution contained (in mmol/L): CsCl 120, EGTA 11, $CaCl_2$ 1 , $MgCl_2$ 5, Na_2-ATP 5, HEPES 10, glucose 11, titrated to pH 7.35 with CsOH. The cells were continuously perfused with extracellular solution contained (in mmol/L): NaCl 35, Choline chloride 100, glucose 10, $CaCl_2$ 1.8, HEPES 10, $MgCl_2$ 1, KCl 5.4, NaH_2PO_4 0.33, $BaCl_2$ 0.1, 4-aminopyridine 5, titrated to pH 7.35 with NaOH. $I_{Ca,\,L}$ activation was measured by applying a 300-ms pulses of voltages between -50 mV and +60 mV in 10 mV steps preceded by a 100-ms prepulse of -50 mV. The steady state inactivation of $I_{Ca,\,L}$ was measured by applying a 300-ms prepulse of potentials from -50 to +60 mV in 10 mV steps, followed by a fixed 300-ms test pulse of +20 mV. Whole cell membrane currents were obtained and assessed with an EPC-9 patch-clamp amplifier (HEKA Electronik, Lambrecht, Germany) in the whole-cell mode by the Pulse/Pulsefit software program. The nonlinear curve fitting of inactivation of $I_{Ca,L}$ was performed with Origin 9.0 (OriginLab Co. USA) using the Boltzmann equation.

References

1 Livak, K. J. & Schmittgen, T. D. Analysis of relative gene expression data using real-time quantitative PCR and the 2(-Delta Delta C(T)) Method. *Methods (San Diego, Calif.)* **25**, 402-408, doi:10.1006/meth.2001.1262 (2001).

2 Zhang, Z. *et al.* Functional roles of Cav1.3(alpha1D) calcium channels in atria: insights gained from gene-targeted null mutant mice. *Circulation* **112**, 1936-1944, doi:10.1161/circulationaha.105.540070 (2005).

3 Qin, M. *et al.* Absence of Rgs5 prolongs cardiac repolarization and predisposes to ventricular tachyarrhythmia in mice. *Journal of molecular and cellular cardiology* **53**, 880-890, doi:10.1016/j.yjmcc.2012.10.003 (2012).

Supplementary Table S1: Anatomic, echocardiographic and hemodynamic parameters in wild-type (WT) and MD1-KO mice at 4 weeks after Sham or aortic banding (AB) operation

Parameters	Sham		AB	
	WT(n = 13)	MD1-KO(n = 13)	WT(n = 13)	MD1-KO(n = 14)
BW(g)	27.07±0.37	27.07±0.49	27.15±0.39	28.21±0.54
HW/BW(mg/g)	4.27±0.03	5.03±0.06	7.68±0.10*	9.26±0.37*#
HW/TL(mg/mm)	6.39±0.08	7.43±0.13	11.20±0.15*	14.20±0.49*#
LW/TL(mg/mm)	7.18±0.14	7.46±0.11	9.15±0.26	14.16±1.56*#
IVSd(mm)	0.74±0.01	0.81±0.01*	0.81±0.02*	0.81±0.02*
IVSs(mm)	1.13±0.02	1.21±0.02	1.25±0.03*	1.21±0.03
LVPWd(mm)	0.74±0.03	0.80±0.02	0.82±0.01*	0.83±0.01*
LVPWs(mm)	1.14±0.02	1.19±0.03	1.26±0.02*	1.24±0.03*
LVEDD(mm)	4.15±0.06	4.16±0.07	5.22±0.07*	5.8±0.10*#
LVESD(mm)	2.45±0.06	2.55±0.09	3.65±0.07*	4.39±0.09*#
FS (%)	40.54±0.98	38.77±1.53	29.77±0.83*	24.07±1.06*#
EF (%)	77.46±1.00	75.23±1.70	63.15±1.31*	53.71±1.85*#
LVESP(mmHg)	109.32±1.51	115.54±3.49	160.86±2.18*	136.46±2.16*#
dp /dt max (mmHg/sec)	10327.00±293.49	10326.62±473.70	7654.39±199.31*	6299.71±219*#
dp/dt min (mmHg/sec)	-9277.23±448.08	-8917.77±258.24	-7233.92±212.19*	-5494.14±331.17*#

BW = body weight; HW = heart weight; LW = lung weight; TL = tibia length; IVSd = interventricular septum diameter in diastole; IVSs = interventricular septum diameter in systole; LVPWd = left ventricular posterior wall diameter in diastole; LVPWs = left ventricular posterior wall diameter in systole; LVEDD = left ventricular end-diastolic diameter; LVESD = left ventricular end-systolic diameter; FS = fractional shortening; EF = ejection fraction; LVESP = left ventricular end-systolic pressure; dp/dt max = the maximum rate of left ventricular pressure development; dp/dt min = the minimum rate of left ventricular pressure development.**P* < 0.05 vs. WT-Sham, #*P* < 0.05 vs. WT-AB. Data are expressed as mean ± SEM.

Supplementary Table S2: The electrophysiological parameters of surface ECG and isolated hearts in WT and MD1-KO mice at 4 weeks after Sham or AB operation

**Parameters**	Sham		AB	
	WT(n = 7–9)	MD1-KO (n = 7–9)	WT (n = 8)	MD1-KO (n = 7–8)
PR interval (ms)	37.96±0.80	40.26±0.65	42.01±3.23	39.61±1.82
RR interval (ms)	125.69±3.85	124.33±6.44	111.64±3.64	102.56±4.00*
QRS duration (ms)	10.43±0.23	11.21±0.46	13.33±0.96	16.97±1.36*#
QTc interval (ms)	52.29±0.68	56.56±2.10	78.40±3.79*	113.44±9.14*#
HR (beats/min)	480.86±15.77	490.90±22.52	543.58±17.19	591.56±24.13*
APD_{90} (ms)	64.87±1.50	70.91±2.19	79.50±4.04*	91.94±3.16*#
threshold interval for APD alternans (ms)	66.43±4.59	78.13±2.49	89.38±2.58*	106.25±4.89*#

QTc interval = corrected QT interval; HR = heart rate; APD = action potential duration; APD_{90} = 90% APD.**P* < 0.03 vs. WT-Sham, #*P* < 0.05 vs. WT-AB. Data are expressed as mean ± SEM.

Supplementary Figure S1

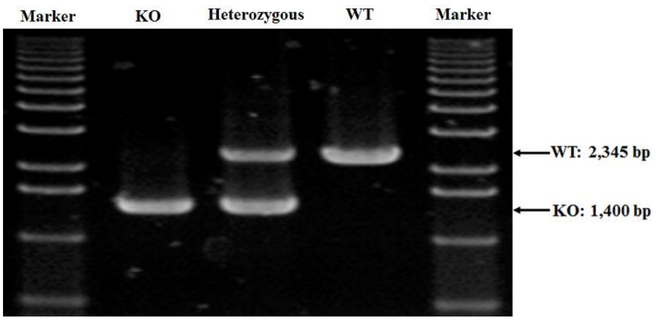

Figure S1: Genotyping of MD1-KO, WT and heterozygous mice using PCR. The amplified PCR product size is 1,400 bp for MD1-KO mice, and 2,345 bp for WT mice.

Supplementary Figure S2

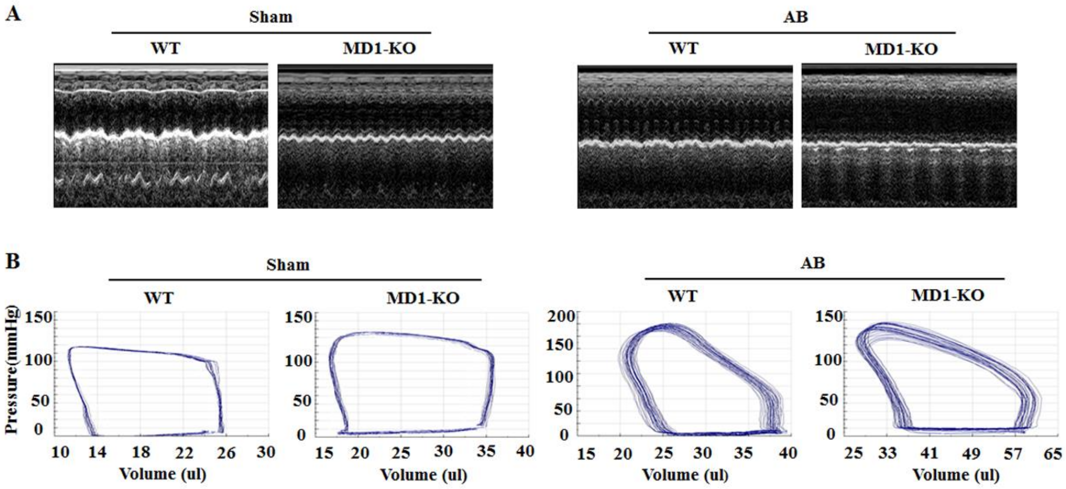

Figure S2: Representative echocardiographic images and pressure-volume (P-V) loop images of the mice hearts. (A) Representative echocardiographic images of the WT and MD1-KO mice at 4 weeks after Sham or AB operation. **(B)** Representative P-V loop images of the WT and MD1-KO mice at 4 weeks after Sham or AB operation.

Supplementary Figure S3

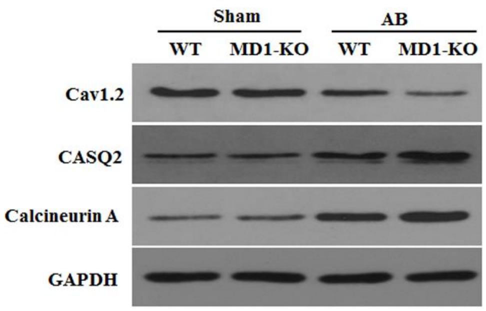

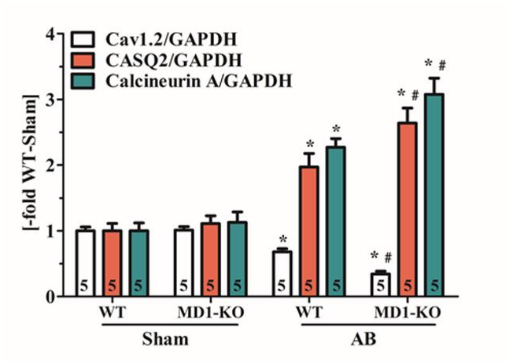

Figure S3: MD1 regulates the expression of Cav1.2, CASQ2, and Calcineurin A in response to pressure overload. Representative western blots (left) and quantitative results (right) showing the expression of Cav1.2, CASQ2, and Calcineurin A in WT and MD1-KO heart samples at 4 weeks after surgery. Number of mice per group are shown inside bars. GAPDH was used as a loading control. *$P < 0.001$ vs. WT-Sham, #$P < 0.01$ vs. WT-AB.

Supplementary Figure S4

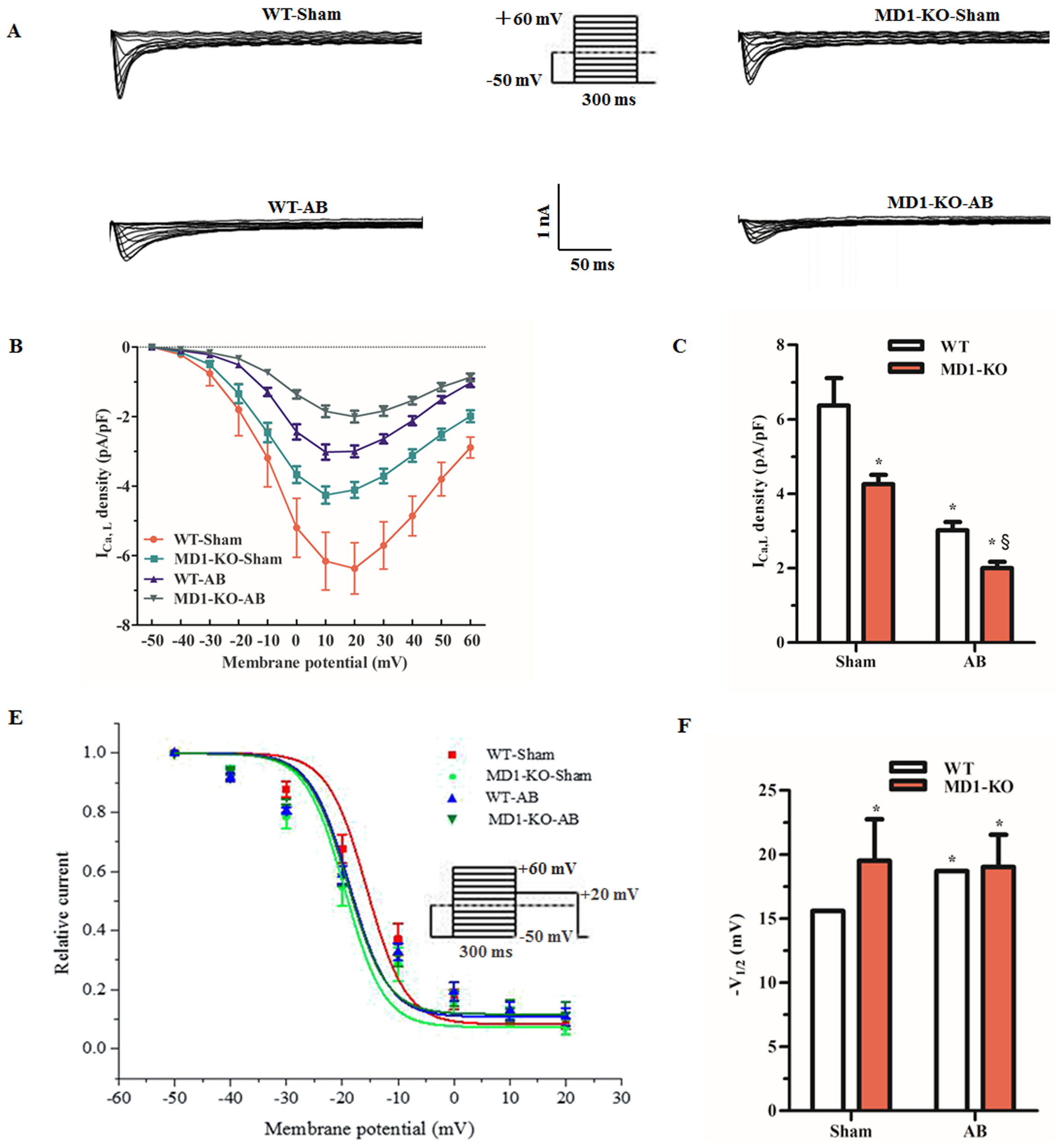

Figure S4: Effects of MD1 absence on $I_{Ca, L}$ in pressure-overloaded mouse hearts. (**A**) Representative current traces of $I_{Ca, L}$ recorded from LV myocytes isolated from WT and MD1-KO mice at 4 weeks after surgery. The pulse protocol is shown at the top of the left panel. (**B**) Current voltage relationships and (**C**) peak current density for $I_{Ca, L}$ of the indicated groups (cells/mice, n = 12–13/5–6). **(D)** Mean values for steady-state inactivation of $I_{Ca, L}$ and **(E)** bar diagram of half-maximal potential ($V_{1/2}$) for $_{Ca, L}$ inactivation of the four groups (cells/mice, n = 12–13/5–6). The pulse protocol is inset in Fig. 5D. Values were fitted to the Boltzmann equation and were analysed with PEMS software. **P* < 0.05 vs. WT-Sham, [§]*P* < 0.005 vs. MD1-KO-Sham.
